# An exercise-associated gut microbiota signature enhances endurance performance: A study combining a human cohort and a mice FMT model

**DOI:** 10.1371/journal.pone.0351316

**Published:** 2026-07-01

**Authors:** Xiongbao Zhang, Yating Fu, Jiahui Chen, Hao Shen, Yumeng Du, Jun Wu, Hongjing Lai, Yu Liu, Jingbo Chen, Xiaomei Hou, Wenjun Chang, Xuefei Hu

**Affiliations:** 1 Faculty of Naval Medicine, Naval Medical University, Shanghai, China; 2 School of Public Health, China Medical University, Shenyang, China; 3 Department of Internal Medicine, Hospital of Naval Marine Corps, Chaozhou, China; Monash University Malaysia, MALAYSIA

## Abstract

**Background:**

The gut microbiota is closely related to exercise, but the interrelationship between the two remains elusive. In this study, we aimed to explore differences in the gut microbiota between young adults with exercise and sedentary lifestyles. In addition, we evaluated the effects of gut microbiota from these different lifestyle populations on endurance exercise capacity.

**Methods:**

The exercise status and nutritional characteristics of young adults were evaluated by PARS-3 and food frequency questionnaires. The gut microbiota of young adults from exercise and sedentary lifestyle groups was analyzed by 16S rRNA analysis. Subsequently, we performed fecal bacteria transplantation (FMT) from the human donors into mice and evaluated the effects on their endurance exercise capacity.

**Results:**

The exercise group exhibited significantly higher gut microbiota diversity compared to the sedentary group. Several beneficial bacteria, including Veillonella, Faecalibacterium, and Bacteroides, were enriched in the exercise group. The FMT experiment confirmed that mice receiving microbiota from the exercise group showed significantly improved endurance exercise capacity.

**Conclusion:**

Young people who exercise regularly possess a more diverse gut microbiota enriched with beneficial bacteria. This exercise-associated microbiota has the potential to directly improve exercise capacity.

## Introduction

Chronic conditions including diabetes, hypertension, cancer, and cardiovascular disease are common, costly, and major causes of death and disability, usually occurred in elder person [1,2]. However, a concerning trend is the rising prevalence and incidence of these conditions among younger adults in many countries, including China [3,4]. To effectively combat the rising prevalence of chronic conditions in young adults, it is vital for clinicians to grasp their underlying drivers and long-term consequences. Among the risk factors, such as genetic predisposition, environmental factors, lifestyle choices, and social determinants of health, sedentary behavior and physical inactivity are one of the critical modifiable risk factors [5,6]. Regular exercise in many cases also has proven to be comparable or superior to drug interventions, in the prevention and management of > 40 chronic conditions [7]. Addressing regular exercise in young adults can help improve well-being and slow disease progression across the life span. However, the exact reasons how exercise training can improve health and slow the progression of chronic diseases are largely unknown.

The gut microbiota is a vast and complex collection of microorganisms that contribute to human health [8] by regulating various physiological functions, such as energy metabolism [9], immune processes [10], and endocrine signaling [11]. Dysbiosis of gut microbiota also have been associated with numerous chronic diseases [8]. Moreover, microbiota transfer experiments in mice suggest a causative role in several non-communicable diseases, including cirrhosis and metabolic disorders [12,13]. Thus, interference of gut microbiota has been a promising way to control or treat chronic diseases. Indeed, it has been reported that exercise can alter the gut microbiome, as a more diverse microbiota was observed in athletes, such as completive cyclist, marathon and rugby, as compared to sedentary controls [14–17]. Interestingly, gut microbiota is also an important determinant in the responsiveness of individuals with chronic diseases such as pre-diabetes to exercise for the improvement of glucose metabolism and insulin sensitivity [18]. Thus, gut microbiota may be critical mediator for the acquiring of health beneficial from regular exercise.

In response to exercise stimuli, gut microbiota adaptation by changing the diversity, composition, and metabolic capacities in a manner that may depend on the intensity, duration, and frequency of the stimuli. Previous studies pay more attention on the population of professional athletes, who usually have experienced physical activities more intensity, duration and frequency. However, whether and how alterations in gut microbiota are functionally involved in the metabolic benefits of exercise in non-athlete younger adults remain obscure. Moreover, the extremes of exercise are usually associated with dietary extremes, which can dramatically modify and confused the alteration of gut microbiota as compared to only exercise. Here, we enrolled 79 college students and investigated the associations between the conditions of physical activities, nutrition diet and diversity and composition of gut microbiota, confirmed the conclusion that regular exercise also significantly improve the gut microbiota and health level in non-athlete young adults.

## Materials and methods

### Human subjects

Volunteers aged 20–25 were recruited for this study with written informed consent. The characteristic information of each subject was collected, including basic information (age, height, weight), exercise habit (Physical activity rating scale 3, PARS-3) [19] and detailed food frequency (Food frequency questionnaire 25, FFQ25). Fecal samples were collected from all participants. To avoid the impact of gender on the research, only male participants were involved in the subsequent studies. Other exclusion criteria: body mass index (BMI) higher than 28 or lower than 18.5 (based on Asian BMI scale), a history of gastrointestinal diseases disorders (e.g., IBS, IBD), prior gastrointestinal surgery, use of medications or probiotics in the preceding 6 months, clinically significant cardiovascular or respiratory conditions, special dietary habits or restrictions, current metabolic or infectious diseases. This study was approved by the Medical Research Ethics Committee of Naval Medical University, and all protocols and processes conformed to the standards of human participants in research as outlined in the Helsinki Declaration. All the questionnaires and specimens were collected during 16/04/2021 ~ 26/04/2021.

### Exercise training and nutritional data collection

The PARS-3, a 3-item measure questionnaire, was used to assess the level of physical activity. In total, there were 3 dimensions measuring exercise intensity, time and frequency, respectively. Each dimension is divided into 5 levels. The formula for calculating amount of physical activity is: amount of physical activity = exercise intensity × exercise time × exercise frequency; thus, the score of physical activity ranges from 0 to 100 points. The scale test-retest reliability is 0.82, and the internal consistency coefficient is 0.75 [20]. Participants were defined to low-exercise (scores range < 30, n = 28), moderate-exercise (scores range 30−43, n = 18) and high-exercise (scores>43, n = 33) according to the distribution of PARS-3 scores (S1a Fig).

Self-reported dietary intake information was collected via a food frequency questionnaire in conjunction with a photographic food atlas. FFQ25, a semi-quantitative food frequency questionnaire, contains 25 food items and previously have been validated in the Chinese population, which was used to asse‌‌ss habitual dietary intake. Participants were asked to recall dietary intakes over the previous 4 weeks. A nutritionist interviewed the subjects for the amount and frequency of the consumption of each food during the latest month. To calculate energy and nutrient intakes, the frequency of consumption was multiplied by the amount consumed for each food item. We measured frequency across nine categories (from “never or almost never” to “≥6 times/day”) and amount across six categories (from “< 50 g” to “> 250 g”). All computations were automated using a program linked to the China Food Composition Tables.

### Fecal sample processing

The fecal samples were collected and stored as previously reported [21,22]. Briefly, fecal samples were defecated into a clean container. Use a sterile sampling spoon to scoop out approximately 2 full spoons (1-3g) of the middle section of the stool that has not been exposed to air or the container. For 16S rRNA sequencing, samples were directly put into the collection tube and be froze to −80°C. For FMT experiment, samples were brought into an anaerobic chamber (70% N_2_, 25% CO_2_, 5% H_2_) after collection. 1 g of the sample was suspended in 15 mL of sterile phosphate buffer supplemented with 0.1% L-cysteine (PBSc). After left standing for 5 min, mixed the supernatant with an equal volume of 40% glycerol in PBSc and stored at −80°C as aliquots (1 mL).

### DNA extraction and 16S rRNA sequencing

Stool samples were stored in a −80°C refrigerator until processed. DNA was prepared using the E.Z.N.A. soil DNA Kit (Omega Bio-tek, Norcross, GA, U.S.) according to manufacturer’s instructions with addition of a bead-beating step. NanoDrop ND-2000 spectrophotometer (Thermo Scientific Inc.) and 1.0% agarose gel electrophoresis was employed to quantify each DNA sample. All DNA specimens were stored at −80°C until to use. The microbiota composition of the samples was established by amplicon sequencing of the hypervariable region V3-V4 of the bacterial 16S rRNA gene. Briefly, the forward primer (5'-ACTCCTACGGGAGGCAGCAG-3') and the reverse primer (5'-GGACTACHVGGGTWTCTAAT-3') were employed for PCR amplification using an ABI GeneAmp 9700 PCR thermocycler (ABI, CA, USA). The amplicons were purified and quantified by the AxyPrep DNA Gel Extraction Kit (Axygen Biosciences, Union City, CA, USA) and Quantus™ Fluorometer (Promega, USA). Purified amplicons were then sequenced on an Illumina MiSeq PE300 platform (Illumina, San Diego, USA) according to the standard protocols. All raw sequencing files are available from the China National Center for Bioinformation (CNCB) database (accession number CRA040381).

### Bioinformatic analysis

Raw reads obtained from sequencing were processed using Cutadapt (Version 1.9.1) to trim and filter barcode and primers. DADA2 (Version 1.22.0) were further used to align the paired-end sequences and perform all the subsequent quality filtering and removing chimeras. Then the optimized sequences were clustered into amplicon sequence variant (ASV) with 97% sequence similarity level. The most abundant sequence for each ASV was selected as a representative sequence. The taxonomy of each ASV representative sequence was analyzed by Naive Bayesian Classifier against the 16S rRNA gene Sliva reference database (Version SSU 138.1) using confidence threshold of 0.7. Based on the ASV information, rarefaction curve and α diversity index including observed index, Chao1 index, ACE index, Shannon index, Simpson index and Fisher index were calculated by MicrobiomeAnalyst. Principal coordinate analysis (PCoA) based on Bray-curtis dissimilarity was adopted in Vegan package (Version 2.5.7) to determine the similarity among microbial communities in different samples. The PERMANOVA test was used to assess the percentage of variation explained by exercise and their statistical significance using Vegan package. Linear discriminant analysis effect size (LEfSe), Wilcoxon Rank-Sum/Signed-Rank Test (Wilcoxon) and Multivariate Association with Linear Models 3 analysis (MaAsLin3) were performed to identify the significantly abundant taxa of bacteria. The ggClusterNet (Version 0.1.0) package and Gephi (Version 0.9.2) were used to construct microbial co-occurrence network to explore the internal community relationships across the samples. If the correlation coefficient of spearman was greater than 0.5 or less than −0.5, and the p value is less than 0.01, the correlation between two nodes is considered to be statistically robust.

### LASSO regression analysis

To select key microbial features associated with exercise status, a LASSO (Least Absolute Shrinkage and Selection Operator) logistic regression analysis was performed. We constructed two separate models at the genus and species levels. The log-transformed relative abundances of taxa served as input variables to predict the binary outcome of exercise groups. A 10-fold cross-validation process was utilized to train the model and tune the penalty parameter (λ). The optimal λ was determined using the one-standard-error (1-se) rule to yield a robust and parsimonious model. Taxa with non-zero coefficients in the final model were identified as potential biomarkers. The performance of the models was assessed by the Area Under the ROC Curve (AUC). The analysis was carried out using the glmnet package in the R studio.

### Animal experiment

The C57BL/6 male mice (specific pathogen-free, 6-weeks old) were purchased from Shanghai Sippe-Bk Lab Animal Co., Ltd. (Experimental Animal License Number SCXK Shanghai, 2013−0016). They were kept under standard conditions (temperature 21 ± 2 °C, humidity 55 ± 5%, 12-h light/dark cycle) with sterile food and water available ad libitum. After an acclimation period of 1 week, mice were treated with 200 μL antibiotic cocktail (ABX) containing ampicillin (1 mg/mL) (Aladdin, Shanghai, China), vancomycin (0.5 mg/mL) (Aladdin, Shanghai, China), neomycin (1 mg/mL) (Aladdin, Shanghai, China), and metronidazole (1 mg/mL) (Sangon Biotech, Shanghai, China) by oral gavage for 5 days. Then, mice were randomly divided into 3 groups (6 mice of each group), and were given 200 μL solvent or the pooled fecal suspension from the selected samples (top 5 high-score individuals and top 5 low-score individuals) by oral gavage once every two days during two weeks. All mice received the running wheel adaptation training for 1 week prior to the exhaustion experiment. Finally, the endurance exercise ability of the mice was assessed by exhaustive exercise. The experiment was terminated for any mouse that was unable to perform the running wheel exercise for three consecutive weeks. The total running distance for the mouse was then calculated for subsequent statistical analysis.

### Statistical analysis

The analysis of study population characteristics and questionnaire information were performed using the IBM SPSS Statistics V.26.0 (IBM, Chicago, IL, United States), GraphPad Prism V.8.3 (La Jolla, California, USA) and R statistical package (Version 4.2.2). The Wilcoxon rank-sum test was used to analyze the changes of different gut microbiota in different classification levels, which limited the prevalence of low count filters >10% and minimum count >2.

## Results

### Association between exercise status and nutritional intake

A total of 79 subjects enrolled in the study, and the characteristics of them, including age, height, weight, BMI, exercise scores, and estimated intakes of nutrition element, were collected. The exercise pattern of the subjects was quite different, as the PARS-3 scores ranged from 9 to 80, which indicated an obvious inter-individual variation (S1a Fig). The association between PARS-3 scores and BMI as well as most of the estimated nutritional elements were not significantly which indicated a slight association between exercise, regular factors and nutrition elements (S1b-c Fig). Based on the distribution of PARS-3 scores and previous cut-off of exercise score, the subjects were classified into 3 groups, containing the groups of low-exercise (scores range < 30, n = 28), moderate-exercise (scores range 30–43, n = 18) and high-exercise (scores>43, n = 33) (S1a Fig). As compared the characteristics among the three groups, the high-exercise population showed higher intakes of calorie, protein (*p* < 0.05), fat, carbohydrate, dietary fiber, cholesterol (*p* < 0.05), vitamin B, vitamin E, and elements such as Ca, Fe and Zn (*p* < 0.05) than the other two groups, which indicated the preference of a high-energy diet for high exercised population (S1 Table).

### Regular Exercise alters the Diversity and Composition of gut microbiota

**Fig 1:**
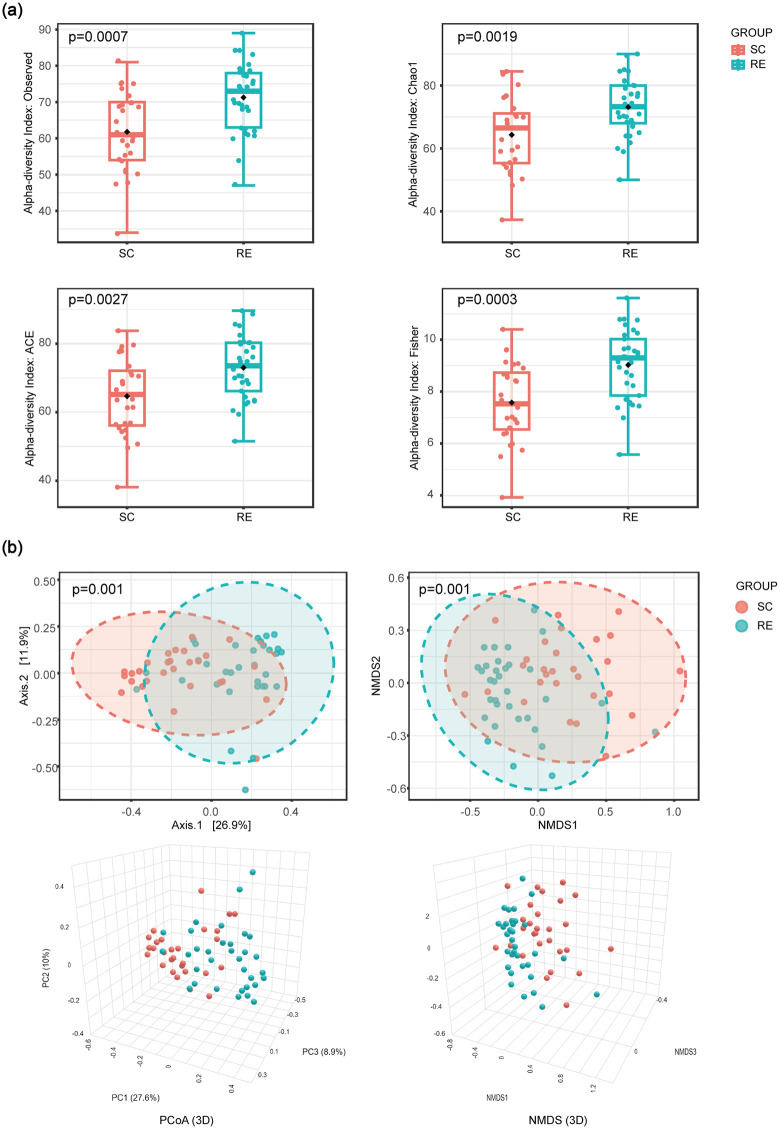
Altered gut microbiota diversity showed significant differences exist in high and low exercise person. **(a)** Observed, Chao1, ACE and Fisher indices were used to estimate the diversity of the gut microbiota. **(b)** Principal coordinate analysis (PCoA) of Bray-Curtis analysis and Non-metric multidimensional scaling (NMDS) demonstrated that individuals from high exercise group were significantly different from low exercise group (p = 0.001, PERMANOVAR). RE: Regular exercise group; SC: Sedentary control group.

**Fig 2:**
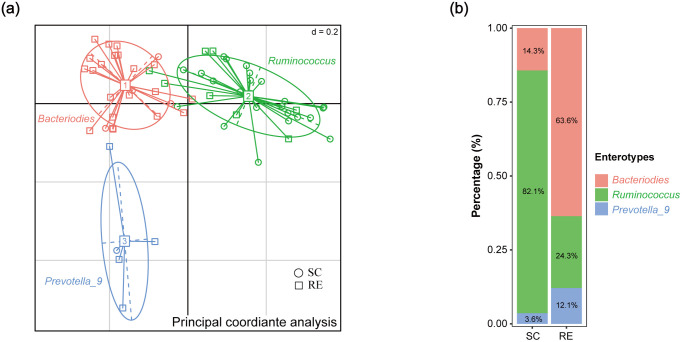
Gut enterotypes in high exercise and low exercise individuals. A total of 61 samples are stratified into three enterotypes. The major contributor in the three enterotypes is Bacteroides, Ruminococcus and Prevotella_9, respectively. **(a)** Three enterotypes were visualized by PCoA of Jensen-Shannon distance at the genus level. **(b)** The proportion of high exercise and low exercise group samples distributed in three enterotypes.

Then we extremely focused on the differences of gut microbiota between the high-exercise group and the low-exercise group. The high-exercise group was defined as the regular exercise population (RE) while the low-exercise group was set as the sedentary control (SC). 61 fecal samples from RE and SC groups were subjected to 16S rRNA sequencing, and a total of 1,772,734 reads were obtained, with an average of 28,554 (±4,980 SD) reads for each RE sample and 29,658 (±5,725 SD) reads for each SC sample. Next, a total of 5024 ASVs were extracted from the reads of all samples, but only 486 ASVs passed the filtering criteria (ASV copy >2, prevalence >10%) for subsequent analysis. At the levels of genus, we found that the alpha-diversity of gut microbiota in RE group was significantly higher than that in SC group (all *P* < 0.05) according to the richness index from multiple methods (Observed species, Chao1 and ACE) and the diversity index (Fisher) (Fig 1a). Furthermore, we calculated the index of beta-diversity by using PCoA based on unweighted Unifrac distance, and found that there were significantly discrete clusters derived from RE and SC groups. This was further confirmed using NMDS method (Fig 1b). Next, we analyzed the gut enterotypes composition in SC and RE groups. As shown in Fig 2, all of the samples are stratified into three enterotypes. The major contributor in the three enterotypes is *bacteroides, ruminococcus* and *prevotella_9* (Fig 2a). The gut enterotypes composition also exhibited significant variations between RE and SC groups: *bacteroides* were extremely more seen while *ruminococcus* showed remarkable decrease in RE groups (Fig 2b).

### Different Co-occurrence pattern between SC and RE populations

**Fig 3:**
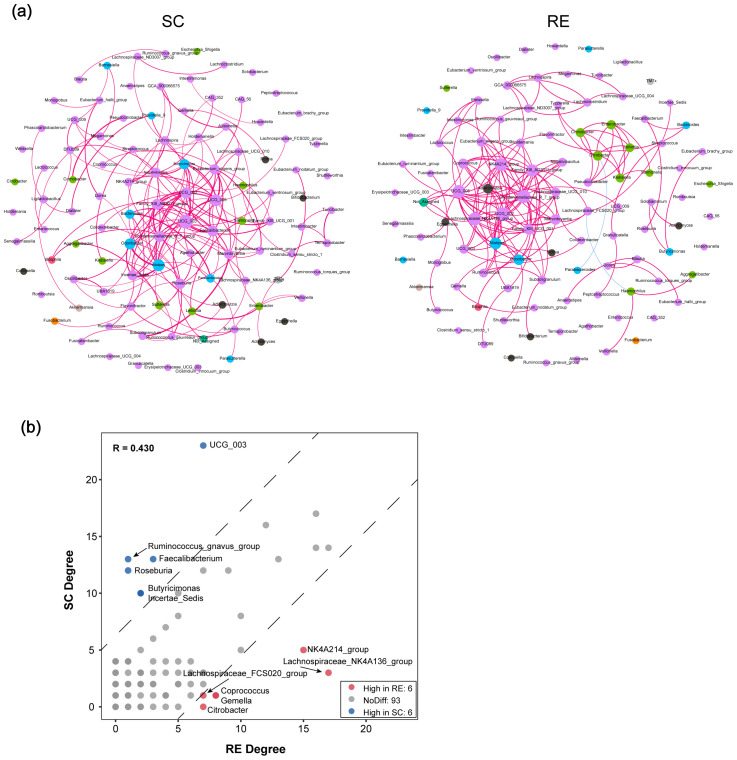
Different Co-occurrence pattern between high and low exercise groups. **(a)** Correlation and Co-occurrence pattern among the 105 genera in high and low exercise groups. Nodes with correlations are labeled with faint red (positive correlation) and blue (negative correlation). Each node represents a different genus, and the size of the nodes indicates its relative abundance. The colors of nodes represent different phylum. Correlations between different genera were determined by the Spearman’s rank correlations analysis. Adjusted P value<0.01. **(b)** Scatter plot of the number of hub node connections in two subnetworks. The horizontal and vertical coordinates represent the number of connecting points in the high-low motion group.

Reads corresponding to 8 phyla, 40 families and 105 genera were detected in 61 samples. Microbial co-occurrence networks were constructed using the 105 genera as nodes and the correlation coefficient (the value < −0.5 or > 0.5, P. adjust < 0.01) of any two genera as edges (Fig 3a). We found the network derived from RE group contained a subnetwork with 75 nodes and 157 edges and another network from SC group contained 86 nodes and 172 edges. Although the parameters of networks, including degree of each node and modules, were almost consistently between 2 subnetworks, however, the nodes and edges of sub-networks from two groups contain different genera (Fig 3b). Importantly, 3 hub nodes from RE network, *Coprococcus, Lachnospiraceae_NK4A136_group* and *Lachnospiraceae_FCS020_group*, have been reported as beneficial bacterium which was not found in SC group, indicating a different organization pattern between the two groups.

### Taxa signatures of gut microbiota driven by exercise training

**Fig 4:**
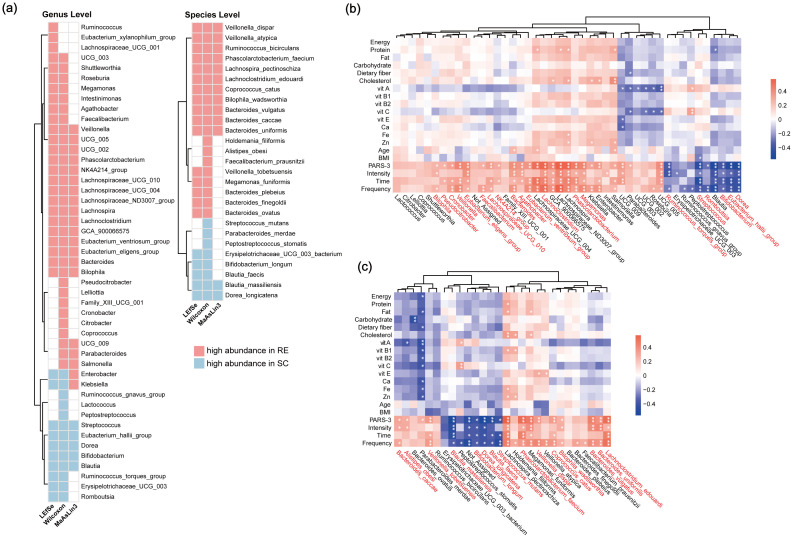
Differentially enriched genera and species. Genera and species can be obtained by both LEfSe (LDA > 3, P. adjust <0.05, corrected FDR < 0.05), Wilcoxon (P. adjust <0.05, corrected FDR < 0.05) and MaAsLin3 (P. adjust <0.05, corrected FDR < 0.05) analysis methods. **(a)** The bacteria marked red are highly enriched in the high exercise group, and the bacteria marked blue are highly enriched in the low exercise group. **(b-c)** The relationship among bacteria enrichment, exercise features and nutritional intake features which were discussed using spearman correlation coefficient at genus and species level, respectively. * p value <0.05; ** p value <0.01.

Next, we compared the taxa proportions of gut microbiota between SC and RE populations by LEfSe, Wilcoxon and MaAsLin3 analysis. The results showed 93 taxa (including 3 phyla, 5 classes, 12 orders, 14 families, 35 genera and 21 species), 121 taxa (including 3 phyla, 8 classes, 15 orders, 22 families, 44 genera and 27 species) and 63 taxa (including 3 phyla, 4 classes, 12 orders, 7 families, 25 genera and 13 species) differently proportions based on LEfSe, Wilcoxon and MaAsLin3 analysis, respectively (S2 and S3 Tables). Consistently, 15 genera and 11 species were identified as higher enrichment in RE group relative to SC group while 5 genera and 2 species were identified as lower enrichment in all three analysis (Fig 4a). Specifically, at least 10 of 16 genera enriched in RE population, such as *Veillonella, Bacteroides,* and *Phascolarctobacterium*, had been reported to have a beneficial effect; inversely, at least 3 of 4 genera reduced in this group including*, Streptococcus* and *Enterobacter*, were identified as one with bad health effect.

Then, to explore exercise related taxon of gut microbiota, based on Wilcoxon analysis, associations among the 45 differently genera, exercise features and nutritional intake features were further discussed using spearman correlation coefficient (r > 0.3 or r < 0.3, P < 0.05) across 61 samples (Fig 4b). We found that there were significant association between 36 of 45 genera with PARS-3 score and/or its factors. Among the 36 genera verified, many were also showed associations with the intake of nutrition elements, such as the intake of protein (*Lachnospiraceae_UCG_004, Lachnospiraceae_ND3007, Lachnospira, Salmonella* and *Balutia*), the intake of cholesterol (*GCA_900066575, Lachnospira, Klebsiella, Enterobacter,* and *Salmonella*) and the intake of Vitamin A and/or C (*UCG_009, Parabacteroides, UCG_005, Roseburia, UCG_002, UCG_003* and *Peptostreptococcus*) which revealed the crosstalk among nutritious feature, exercise and gut microbiota. More importantly, 20 genera tend to be affected by exercise independently, including several exercises educated genera (*Veillonella, Phascolarctobacterium, Lachnospira* and *Faecalibacterium*), which indicated the strong relationship between gut microbiota and exercise. As considering the level of species, 18 of 28 differently enriched species were verified as exercise related items which were significantly associated with PARS-3 score (Fig 4c). Among the 18 species, 3 species (*Parabacteroides_merdae, Lachnospira_pectinoschiza,* and *Megamonas_funiformis*) were significantly associated with the intakes of nutrition elements as well while the other 15 species tended to be independently associated with exercise situation.

### Fecal microbiome transplant of high-exercise individuals enhancing exercise performance in mice

Next, we focused on the 20 genus and 15 species which were identified as independently associated with PARS-3 scores. While their individual ROC curves and AUC values confirmed their ability to effectively distinguish between the RE and SC groups (S2a Fig), we sought to build a more powerful model for predicting PARS-3 score. To this end, we employed a LASSO (Least Absolute Shrinkage and Selection Operator) regression model for refined feature selection and model construction. Using 10-fold cross-validation, we identified the optimal penalty parameter (λ = 0.1188 and 0.0595 at genus and species level, respectively) based on the minimum criteria plus the one-standard-error rule, which yielded a robust and parsimonious model. 9 key taxa at both the genus and species levels were selected and 2 corresponding models were conducted. The two multi-variable models demonstrated a significantly improved ability to discriminate between the RE and SC groups compared to any single taxon, achieving AUCs of 0.92 and 0.94, respectively. This suggests their potential as robust composite biomarkers for assessing an individual’s exercise status (S2b-c Fig).

**Fig 5:**
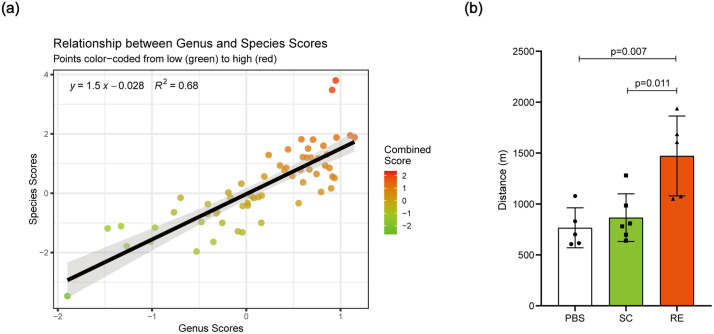
Fecal microbiome transplant enhancing exercise performance. **(a)** Scatter plot of all samples based on 2 taxa signatures. The linear fitting of the two groups is consistent. **(b)** The running distance of three groups of mice inoculated with different sources of fecal microbiome in the exhaustion experiment.

Subsequently, we employed fecal microbiome transplant (FMT) to experimentally verify the effect of the models. The 61 samples in our cohort were rescored based on the two models. We ranked the new scores of each sample and founded that the 2 models predicted the similar results (Fig 5a). Then, stools from the top 5 high-score individuals and top 5 low-score individuals were used to carried out fecal microbiome transplant (FMT) based on ABX-treated mice model. After 2 weeks of FMT and 1 week of running wheel training, all groups of the mice were subjected to perform exhaustion test to exhibite the impact on exercise endurance. The results showed that mice which received high-score fecal samples had the strongest running capacities with more than 1.7 times running distance compared with the other 2 groups (Fig 5b). This revealed that the two models accurately predicted the exercise status of individuals, in addition, the gut microbiota of the high-exercise individuals had the capacity to improve the exercise performance of ordinary individuals. The signatures constructed in this study could be served as predictor and potential target for running capacities promoting.

## Discussion

This study investigated the diversity and composition of the gut microbiota of college students who were sedentary or underwent regular exercise. The results confirmed the enhancement of microbial diversity and increasing abundance of part beneficial bacterium in regularly exercise group compared with sedentary control, which supporting previous insights into the beneficial influence of physical exercise on the diversity and composition of the gut microbiota.

In this study, the diversity of gut microbiota in RE group was significantly higher than that in SC group matched by age, height and weight, and the principal coordinate analysis showed that there were clustering differences between the two groups, which were consistent with the results of previous studies on competitive athletes. Diversity is an important index to evaluate the stability and performance of microbial community [23]. In principle, a more diverse gut microbiota is thought to be beneficial, because the lack of gut microbiota diversity has been associated with multiple diseases, including obesity, inflammatory bowel disease (IBD), depression, and diabetes. A more diverse microbiota is thought to enhance metabolism and be highly resistant against harmful environmental factors, involving nutrient absorption [23], energy acquisition [24], inflammation regulation [25] and host immune response [26]. Compared with SC group, the proportion of several gut microbial taxa at the phylum level also changed in RE group, including *Bacteroidetes* and *Firmicutes*. Interestingly, Bacteroidetes and Firmicutes are two phyla with the highest abundance of gut microbiota and containing the vast majority of beneficial bacterium, and more beneficial bacteria tend to be positively associated with health [27], which further confirmed that exercise can promote health and reduce the risk of disease in previous studies.

The Wilcoxon and LEfSe methods were used to find the differential microbiota between the two groups in this study, we found that there was a large overlap between the results obtained by the two methods, indicating that the analysis results were relatively stable and the results had high confidence. The overlap included 32 genera and 21 species, of which 22 genera and 16 species were elevated in RE and 10 genera and 5 species were elevated in SC. It is worth noting that among the increased bacteria in the exercise group, many were reported to be beneficial to health. For example, *Bacteroides* was the principal synthesizer of vitamin K, which could prevent or treat osteoporosis by increasing bone mineral density [28]. They were also primary producers of short-chain fatty acids in the human gut, mainly in the form of propionate and butyrate. Propionate played an anti-inflammatory role by inducing apoptosis of human colon cancer cells [29] while butyrate increased the expression of tight-junction proteins in the gut to reduce the potential intestinal hyperpermeability [30]. *Roseburia* could produce SCFAs, especially butyrate, which affect gut movement, maintain immunity and anti-inflammatory properties, and reduce the occurrence of inflammatory bowel disease, type 2 diabetes, atherosclerosis [31]. Meanwhile, the high abundance of *Klebsiella*, *Streptococcus* and *Enterobacter* in the sedentary group was identified as having adverse health effects [32,33]. Therefore, the differential bacteria found in this study were significantly associated with health and disease, and exercise improved the composition and structure of the gut microbiota, promoted the abundance of beneficial bacteria, while sedentary behavior promoted the growth of harmful bacteria.

It was worthy mention that in addition to healthy exercise that can improve the gut microbiota, diet is also one of the keys to affecting the gut microbiota [34]. Members of the gut microbiota are not only sensitive to specific dietary components but also respond differently to nutrition in countless temporal and geographical contexts. Modulation of the gut microbiota composition and function by the diet could resulted in beneficial or detrimental consequences on host health. For this reason, we excluded gut microbiota related to diet in order to obtain exercise-driven gut microbiota independent of diet. Finally, there were 20 genera and 15 species were identified to be affected by exercise independently including 14 genera, 10 species abundant in RE group and 6 genera, 5 species abundant in SC group.

SCFAs producing microbiome were found multiple times in 2 exercise educated taxa signatures (20 genera signature and 15 species signature) in our study, *Lachnospiraceae, Megamonas, Phascolarctobacterium, Lachnospira, Veillonella, Bacteroides* and *Faecalibacterium* were participate in the fermentation process of intestinal contents to form SCFAs. SCFAs is a key regulatory factor between gut microbiota and the metabolism of the body’s motor organs [35]. They can participate in the body’s energy metabolism, promote mitochondrial synthesis, enhance tricarboxylic acid cycle, increase glucose uptake and fatty acid oxidation in skeletal muscle, inhibit lipid deposition in skeletal muscle, and improve the quality of skeletal muscle. In addition, the acetate in SCFAs can be used as an important substrate for endurance exercise, providing energy for the body and enhancing endurance exercise ability. Propionate can act on peripheral organs and tissues that express SCFA receptors to improve endurance exercise ability. Butyrate can improve gut inflammation and oxidation status, provide energy for intestinal epithelial cells, protect normal intestinal function and indirectly improve endurance exercise ability [36,37]. We also found the high abundance of *Veillonella* in the RE group. *Veillonella* can metabolize lactic acid produced during exercise and produce SCFAs which were absorbed by skeletal muscles to increase the maximum oxygen consumption and enhance exercise ability. It could also produce NO through the inorganic nitrate pathway, improve muscle strength and mitochondrial efficiency, and thus improve exercise endurance [38]. Moreover, *in vivo* experiments in this study further confirmed that transplanting fecal bacteria from populations with a high abundance of taxa signatures can indeed enhance motor performance of mice, which further demonstrating the close relationship between exercise and gut microbiota. It is speculative but not implausible that exercise, independent of dietary factors, confers observable changes in the gut microbiota that give the host a healthier state and vitality.

There were still limitations in this exploratory study due to the lack of in-depth dietary analysis and a matching regular exercise cohort with low physical activity. In future studies, we will increase another regular exercise cohort with low physical activity to reduce the influence of daily high-intensity physical work on the result, and strictly control for diet and exercise variables, since diet plays a role alongside with exercise in influencing the composition and abundance of human gut microbiota. Although due to the limitations of 16S rRNA sequencing technology, we could only focus on genera classification analysis, targeting a limited number of species, which may lose potentially beneficial species, the experimental data here provide valuable clues about the potential motion-enhancing effects of gut microbiota. The use of technologies such as metagenomic sequencing will helps us to further delineate a more comprehensive map of the gut microbiota related to exercise. In addition, this study was limited to a male-only cohort, which restricts the generalizability of our findings to females. Future research incorporating a female cohort is warranted to validate these results and to explore the interplay between gender, exercise status, and the gut microbiota.

## Conclusion

In conclusion, our study suggests that regular exercise may reshapes the human gut microbiota, potentially fostering a community characterized by higher alpha-diversity, a distinct composition, and a more stable co-occurrence network. We identified specific microbial signatures that appear to be independently associated with exercise. Through fecal microbiota transplantation (FMT), we further tested the effect of the specific microbial signatures on prolonging exercise endurance in mice. These findings highlight the gut microbiota as a key mediator of the physiological benefits of exercise. The identified microbial taxa could serve as potential biomarkers for exercise adaptation and may represent promising targets for novel interventions aimed at improving athletic performance and promoting health in sedentary individuals.

## Supporting information

S1 Fig The PARS-3 scores of the subjects and its relationship among physical indicators and nutritional intake. (a) The distribution of PARS-3 scores of the 79 individuals and the classification of the 3 groups of low-exercise, middle-exercise and high- exercise. (b-c) The correlation heatmap showing the associations among PARS-3 score, BMI and nutritional intake features of the individuals. Spearman’s rank correlation analysis, * p value <0.05; ** p value <0.01.
(TIF)

S2 Fig Construction and validation of microbial biomarker models for assessing exercise status. (a) Receiver operating characteristic (ROC) curves for the enrichment of 20 genera and 15 species independently associated with PARS-3 scores. (b-c) Least Absolute Shrinkage and Selection Operator (LASSO) regression analysis at the genus level and species level.
(TIF)

S1 Table Body characteristics and estimated intakes of nutrition elements of subjects among 3 exercise groups. (DOCX)

S2 Table Taxa proportions in Phylum, Class, Order and Family levels of gut microbiota between SC and RE populations by LEfSe, Wilcoxon and MaAsLin3 analysis. (DOCX)

S3 Table Taxa proportions in genus and species level of gut microbiota between SC and RE populations by LefSe, Wilcoxon and MaAsLin3 analysis. (DOCX)
