# Supplementary figures and images for: An exercise-associated gut microbiota signature enhances endurance performance: A study combining a human cohort and a mice FMT model
Source: PLoS One. 2026 Jul 1;21(7):e0351316. doi: 10.1371/journal.pone.0351316 (PMC13322530; doi:10.1371/journal.pone.0351316)

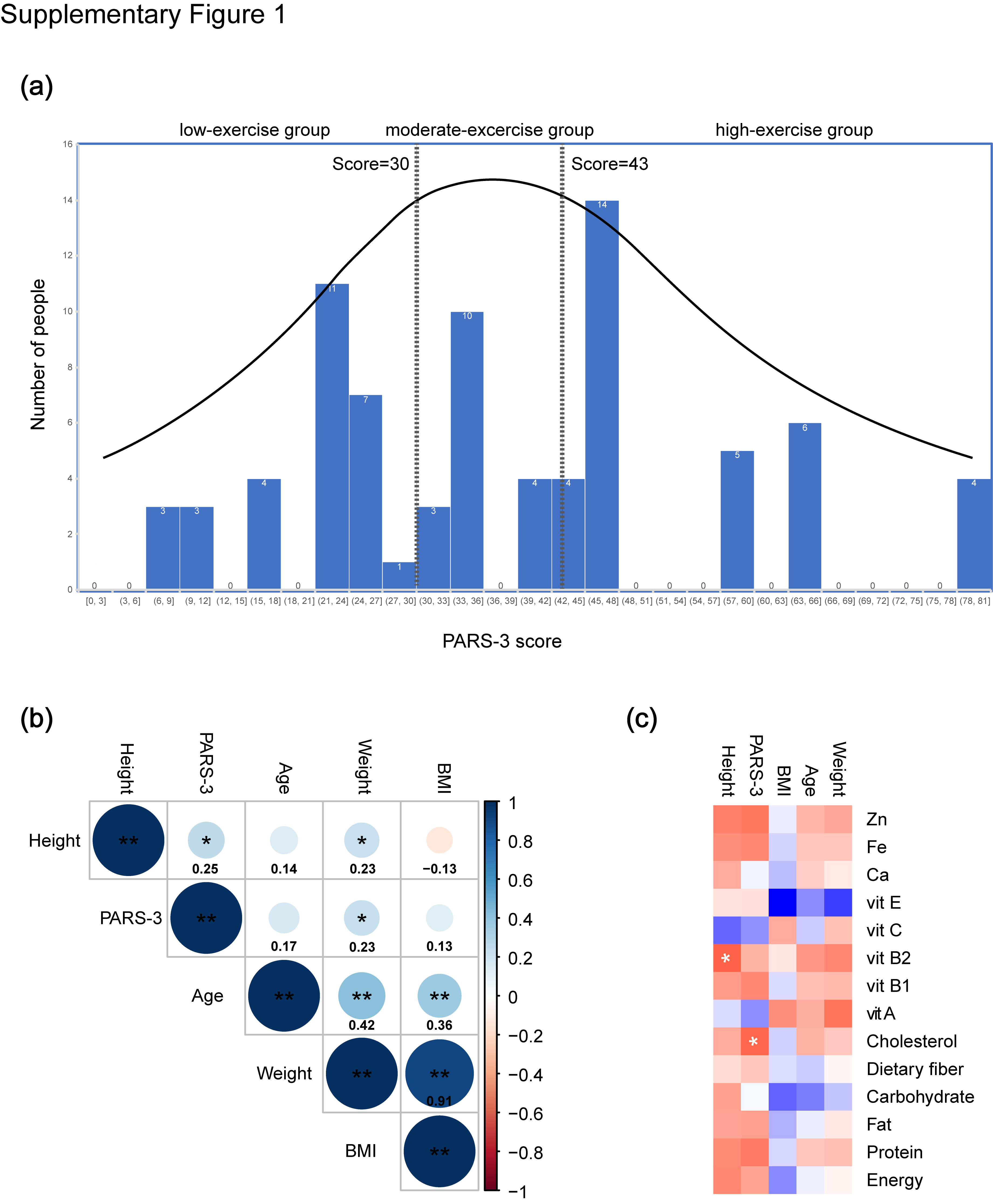

Supplement: S1 Fig — (a) The distribution of PARS-3 scores of the 79 individuals and the classification of the 3 groups of low-exercise, middle-exercise and high- exercise. (b-c) The correlation heatmap showing the associations among PARS-3 score, BMI and nutritional intake features of the individuals. Spearman’s rank correlation analysis, * p value <0.05; ** p value <0.01. (TIF) [file pone.0351316.s001.tif]

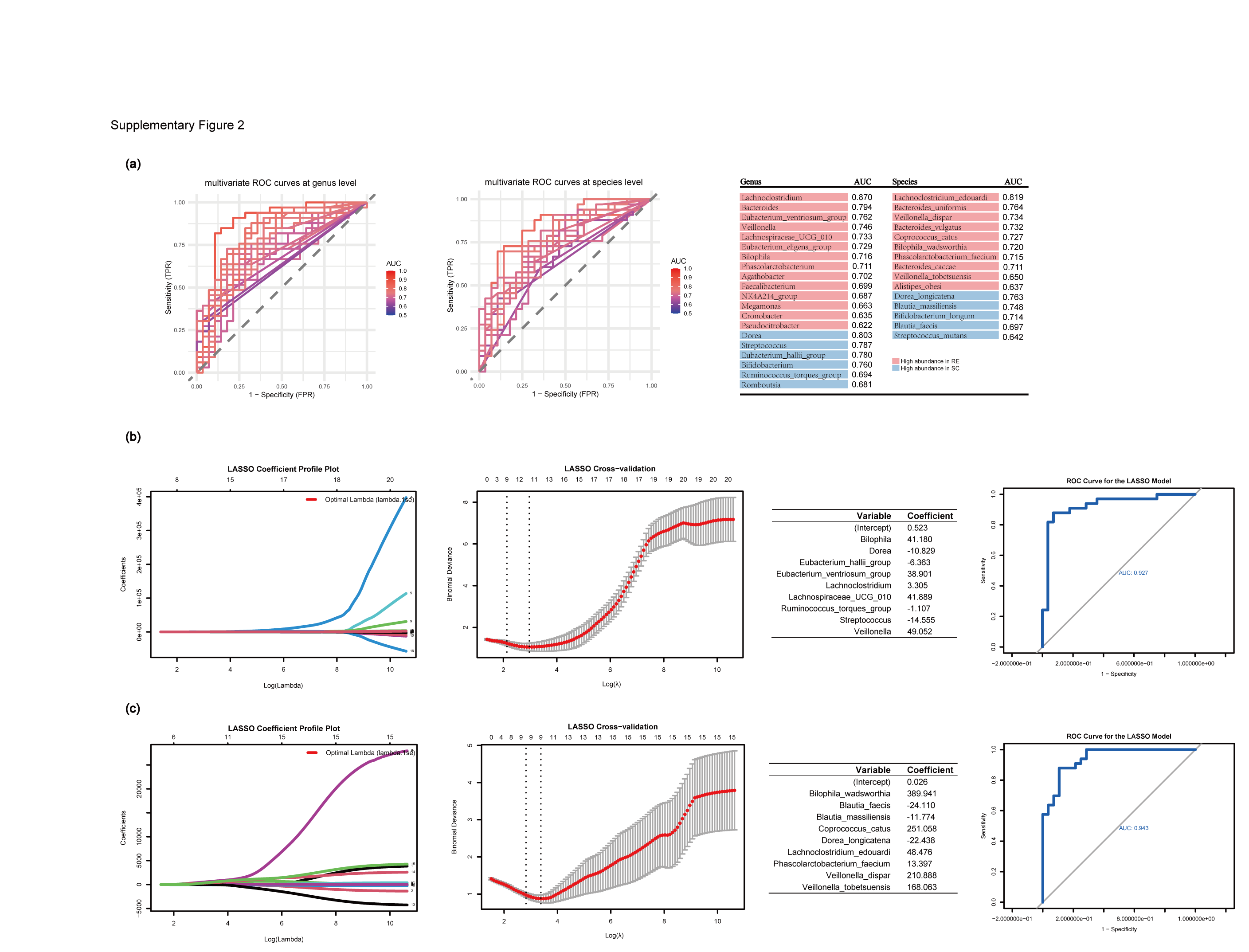

Supplement: S2 Fig — (a) Receiver operating characteristic (ROC) curves for the enrichment of 20 genera and 15 species independently associated with PARS-3 scores. (b-c) Least Absolute Shrinkage and Selection Operator (LASSO) regression analysis at the genus level and species level. (TIF) [file pone.0351316.s002.tif]
