# Supplementary material for: An exercise-associated gut microbiota signature enhances endurance performance: A study combining a human cohort and a mice FMT model
Source: PLoS One. 2026 Jul 1;21(7):e0351316. doi: 10.1371/journal.pone.0351316 (PMC13322530; doi:10.1371/journal.pone.0351316)
Supplement: S1 Table — (DOCX) [file pone.0351316.s003.docx]

Supplementary Table 1. Body characteristics and estimated intakes of nutrition elements of subjects among 3 exercise groups

| Factors | **Total (N=79)** | **High-exercise (n=33)** | **Moderate-exercise (n=18)** | **Low-exercise (n=28)** | **P1 value** | **P2 value** |
| --- | --- | --- | --- | --- | --- | --- |
| **Age** (years) | 21.4 (2.0) | 21.5 (1.8) | 21.9 (2.2) | 20.8 (1.9) | 0.452 | 0.126 |
| **Height** (cm) | 180.1 (3.7) | 181.2 (3.9) | 180.7 (3.1) | 178.5 (3.3) | 0.700 | **0.006** |
| **Weight** (kg) | 73.6 (8.5) | 75.6 (9.2) | 73.3 (7.2) | 71.3 (8.0) | 0.361 | 0.064 |
| **BMI** (kg/m^2^) | 22.7 (2.5) | 23.0 (2.5) | 22.5 (2.3) | 22.4 (2.6) | 0.434 | 0.356 |
| **PARS-3** | 39.1 (17.7) | 56.2 (11.4) | 35.9 (2.9) | 21.0 (7.1) | **<0.001** | **<0.001** |
| **Intensity** (%) |  |  |  |  | 0.072 | **<0.001** |
| Low | 6 (7.6) | 0 (0.0) | 0 (0.0) | 6 (21.4) |  |  |
| Moderate | 33 (41.8) | 7 (21.2) | 9 (50.0) | 17 (60.7) |  |  |
| High | 40 (50.6) | 26 (78.8) | 9 (50.0) | 5 (17.9) |  |  |
| **Time** (%) |  |  |  |  | **<0.001** | **<0.001** |
| 11-20 (min) | 3 (3.8) | 0 (0.0) | 0 (0.0) | 3 (10.7) |  |  |
| 21-30 (min) | 22 (27.9) | 0 (0.0) | 7 (38.9) | 15 (53.6) |  |  |
| 31-59 (min) | 37 (46.8) | 18 (54.5) | 9 (50.0) | 10 (35.7) |  |  |
| >60 (min) | 17 (21.5) | 15 (45.5) | 2 (11.1) | 0 (0.0) |  |  |
| **Frequency** (%) |  |  |  |  | 0.301 | **<0.001** |
| 2-3/month | 1 (1.2) | 0 (0.0) | 1 (5.6) | 0 (0.0) |  |  |
| 1-2/week | 27 (34.2) | 2 (6.1) | 3 (16.7) | 22 (78.6) |  |  |
| 3-5/week | 33 (41.8) | 18 (54.5) | 9 (50.0) | 6 (21.4) |  |  |
| 1/day | 18 (22.8) | 13 (39.4) | 5 (27.8) | 0 (0.0) |  |  |
| **Energy** (kcal) | 1772.3 (1134.3) | 2062.3 (1298.9) | 1525.08 (1165.44) | 1589.36 (827.03) | 0.150 | 0.108 |
| **Protein** (g) | 84.7 (59.1) | 102.3 (70.3) | 72.72 (56.85) | 71.83 (39.59) | 0.133 | **0.047** |
| **Fat** (g) | 66.4 (47.6) | 80.3 (57.1) | 55.03 (40.25) | 57.44 (35.35) | 0.103 | 0.118 |
| **Carbohydrate** (g) | 216.8 (139.8) | 240.5 (157.0) | 193.71 (159.85) | 203.56 (99.57) | 0.317 | 0.287 |
| **Dietary fiber** (g) | 9.3 (7.4) | 10.4 (8.4) | 8.91 (8.41) | 8.15 (5.41) | 0.536 | 0.241 |
| **Cholesterol** (mg) | 240.8 (199.4) | 312.8 (233.9) | 195.23 (177.53) | 185.39 (138.62) | 0.069 | **0.014** |
| **vit A** (mg) | 538.2 (398.4) | 519.56 (304.85) | 551.45 (594.84) | 551.73 (353.32) | 0.800 | 0.704 |
| **vit B1** (mg) | 0.9 (0.6) | 1.05 (0.75) | 0.74 (0.62) | 0.70 (0.40) | 0.151 | 0.073 |
| **vit B2** (mg) | 1 (0.7) | 1.20 (0.73) | 0.97 (0.81) | 0.92 (0.48) | 0.309 | 0.083 |
| **vit C** (mg) | 61.3 (57.3) | 59.33 (49.99) | 66.46 (80.29) | 60.39 (49.33) | 0.697 | 0.874 |
| **vit E** (mg) | 17.1 (16.9) | 20.75 (22.24) | 13.76 (10.56) | 14.88 (11.75) | 0.215 | 0.160 |
| **Ca** (mg) | 417.3 (301.7) | 466.86 (371.81) | 377.47 (287.61) | 384.38 (204.50) | 0.381 | 0.311 |
| **Fe** (mg) | 17.2 (12.3) | 20.41 (14.52) | 15.33 (13.08) | 14.69 (7.60) | 0.223 | 0.051 |
| **Zn** (mg) | 11.5 (8.1) | 14.06 (9.86) | 9.82 (7.63) | 9.54 (5.06) | 0.120 | **0.033** |

Quantitative data shown as mean (SD) and classified data shown as count(percentage). T-test was used to compare age, weight, height, BMI, PARS-3, energy, fat, dietary fiber, vit B1, vit C, vit E, Ca and Fe. Fisher test was used to compare classification data intensity, time and frequency. Intensity indicates the intensity of each exercise. Time indicates the time of each exercise. Frequency indicates the number of exercises in a fixed time range. Non-parametric test was used to compared the others. P1 value: The high exercise group was compared with the moderate exercise group. P2 value: The high exercise group was compared with the low exercise group.
