# Supplementary material for: An exercise-associated gut microbiota signature enhances endurance performance: A study combining a human cohort and a mice FMT model
Source: PLoS One. 2026 Jul 1;21(7):e0351316. doi: 10.1371/journal.pone.0351316 (PMC13322530; doi:10.1371/journal.pone.0351316)
Supplement: S2 Table — (DOCX) [file pone.0351316.s004.docx]

Supplementary Table 2: Taxa proportions in Phylum, Class, Order and Family levels of gut microbiota between SC and RE populations by LEfSe, Wilcoxon and MaAsLin3 analysis.

|  | **Differential taxa** | **LEfSe analysis** | | **Wilcox analysis** | | **MaAsLin3** | |
| --- | --- | --- | --- | --- | --- | --- | --- |
|  |  | **RE** | **SC** | **RE** | **SC** | **RE** | **SC** |
| Phylum | Actinobacteriota |  | ✓ |  | ✓ |  | ✓ |
|  | Bacteroidota | ✓ |  | ✓ |  | ✓ |  |
|  | Desulfobacterota | ✓ |  | ✓ |  | ✓ |  |
| Class | Actinobacteria |  | ✓ |  | ✓ |  | ✓ |
|  | Bacilli |  | ✓ |  | ✓ |  | ✓ |
|  | Bacteroidia | ✓ |  | ✓ |  | ✓ |  |
|  | Clostridia |  |  |  | ✓ |  |  |
|  | Coriobacteriia |  |  |  | ✓ |  |  |
|  | Desulfovibrionia | ✓ |  | ✓ |  | ✓ |  |
|  | Negativicutes | ✓ |  | ✓ |  |  |  |
|  | Saccharimonadia |  |  |  | ✓ |  |  |
| Order | Acidaminococcales | ✓ |  | ✓ |  | ✓ |  |
|  | Bacteroidales | ✓ |  | ✓ |  | ✓ |  |
|  | Bifidobacteriales |  | ✓ |  | ✓ |  | ✓ |
|  | Burkholderiales | ✓ |  | ✓ |  | ✓ |  |
|  | Clostridiales |  |  |  | ✓ |  |  |
|  | Coriobacteriales |  |  |  | ✓ |  |  |
|  | Desulfovibrionales | ✓ |  | ✓ |  | ✓ |  |
|  | Erysipelotrichales |  | ✓ |  | ✓ |  | ✓ |
|  | Lachnospirales |  | ✓ |  | ✓ |  | ✓ |
|  | Lactobacillales |  | ✓ |  | ✓ |  | ✓ |
|  | Monoglobales |  |  |  | ✓ |  |  |
|  | Peptostreptococcales_Tissierellales |  | ✓ |  | ✓ |  | ✓ |
|  | Saccharimonadales |  |  |  | ✓ |  |  |
|  | Staphylococcales |  |  |  | ✓ |  |  |
|  | Veillonellales_Selenomonadales | ✓ |  | ✓ |  | ✓ |  |
|  | Clostridia_UCG_014 | ✓ |  |  |  | ✓ |  |
|  | Oscillospirales | ✓ |  |  |  | ✓ |  |
| Family | Acidaminococcaceae | ✓ |  | ✓ |  | ✓ |  |
|  | Bacteroidaceae | ✓ |  | ✓ |  | ✓ |  |
|  | Bifidobacteriaceae |  | ✓ |  | ✓ |  | ✓ |
|  | Carnobacteriaceae |  |  |  | ✓ |  |  |
|  | Clostridiaceae |  |  |  | ✓ |  |  |
|  | Coriobacteriaceae |  |  |  | ✓ |  |  |
|  | Desulfovibrionaceae | ✓ |  | ✓ |  | ✓ |  |
|  | Enterococcaceae |  |  |  | ✓ |  |  |
|  | Erysipelatoclostridiaceae |  | ✓ |  | ✓ |  |  |
|  | Eubacterium_coprostanoligenes_group | ✓ |  | ✓ |  |  |  |
|  | Gemellaceae |  |  |  | ✓ |  |  |
|  | Lachnospiraceae |  | ✓ |  | ✓ |  |  |
|  | Lactobacillaceae |  |  |  | ✓ |  |  |
|  | Monoglobaceae |  |  |  | ✓ |  |  |
|  | Oscillospiraceae | ✓ |  | ✓ |  | ✓ |  |
|  | Peptostreptococcaceae |  | ✓ |  | ✓ |  |  |
|  | Ruminococcaceae | ✓ |  |  |  |  |  |
|  | Saccharimonadaceae |  |  |  | ✓ |  |  |
|  | Selenomonadaceae | ✓ |  | ✓ |  | ✓ |  |
|  | Streptococcaceae |  | ✓ |  | ✓ |  |  |
|  | Sutterellaceae | ✓ |  | ✓ |  |  |  |
|  | Tannerellaceae |  |  |  | ✓ |  |  |
|  | Veillonellaceae | ✓ |  | ✓ |  | ✓ |  |
