# Supplementary material for: An exercise-associated gut microbiota signature enhances endurance performance: A study combining a human cohort and a mice FMT model
Source: PLoS One. 2026 Jul 1;21(7):e0351316. doi: 10.1371/journal.pone.0351316 (PMC13322530; doi:10.1371/journal.pone.0351316)
Supplement: S3 Table — (DOCX) [file pone.0351316.s005.docx]

Supplementary Table 3. Taxa proportions in genus and species level of gut microbiota between SC and RE populations by LefSe, Wilcoxon and MaAsLin3 analysis.

| **Differential taxa** | **LEfSe analysis** | | **Wilcox analysis** | | **MaAsLin3** | | **Relative abundance** | |
| --- | --- | --- | --- | --- | --- | --- | --- | --- |
|  | **RE** | **SC** | **RE** | **SC** | **RE** | **SC** | **RE** | **SC** |
| **Genus** |  |  |  |  |  |  |  |  |
| Agathobacter | ✓ |  | ✓ |  |  |  | 0.050336 | 0.025618 |
| Bacteroides | ✓ |  | ✓ |  | ✓ |  | 0.083930 | 0.033150 |
| Bifidobacterium |  | ✓ |  | ✓ |  | ✓ | 0.042918 | 0.089269 |
| Bilophila | ✓ |  | ✓ |  | ✓ |  | 0.000612 | 0.000057 |
| Blautia |  | ✓ |  | ✓ |  | ✓ | 0.057816 | 0.184659 |
| Citrobacter |  |  | ✓ |  |  |  | 0.002923 | 0.001215 |
| Coprococcus |  |  | ✓ |  |  |  | 0.014911 | 0.007266 |
| Cronobacter |  |  | ✓ |  |  |  | 0.000108 | 0.000006 |
| Dorea |  | ✓ |  | ✓ |  | ✓ | 0.012709 | 0.038785 |
| Enterobacter |  | ✓ |  | ✓ | ✓ |  | 0.007564 | 0.016326 |
| Erysipelotrichaceae_UCG_003 |  | ✓ |  | ✓ |  |  | 0.008276 | 0.012402 |
| Eubacterium_eligens_group | ✓ |  | ✓ |  | ✓ |  | 0.004305 | 0.001967 |
| Eubacterium_hallii_group |  | ✓ |  | ✓ |  | ✓ | 0.013406 | 0.039165 |
| Eubacterium_ventriosum_group | ✓ |  | ✓ |  | ✓ |  | 0.003147 | 0.001188 |
| Eubacterium_xylanophilum_group | ✓ |  |  |  |  |  | 0.000607 | 0.000002 |
| Faecalibacterium | ✓ |  | ✓ |  |  |  | 0.141999 | 0.067950 |
| Family_XIII_UCG_001 |  |  | ✓ |  |  |  | 0.000368 | 0.000100 |
| GCA_900066575 | ✓ |  | ✓ |  | ✓ |  | 0.000288 | 0.000061 |
| Intestinimonas | ✓ |  | ✓ |  |  |  | 0.000898 | 0.000209 |
| Klebsiella |  | ✓ |  | ✓ | ✓ |  | 0.019452 | 0.025771 |
| Lachnoclostridium | ✓ |  | ✓ |  | ✓ |  | 0.020569 | 0.005273 |
| Lachnospira | ✓ |  | ✓ |  | ✓ |  | 0.011288 | 0.001567 |
| Lachnospiraceae_ND3007_group | ✓ |  | ✓ |  | ✓ |  | 0.002196 | 0.000279 |
| Lachnospiraceae_UCG_001 | ✓ |  |  |  |  |  | 0.000622 | 0.000228 |
| Lachnospiraceae_UCG_004 | ✓ |  | ✓ |  | ✓ |  | 0.002522 | 0.000744 |
| Lachnospiraceae_UCG_010 | ✓ |  | ✓ |  | ✓ |  | 0.001447 | 0.000293 |
| Lactococcus |  |  |  | ✓ |  |  | 0.002980 | 0.003020 |
| Lelliottia |  |  | ✓ |  |  |  | 0.000354 | 0.000225 |
| Megamonas | ✓ |  | ✓ |  |  |  | 0.067919 | 0.017580 |
| NK4A214_group | ✓ |  | ✓ |  | ✓ |  | 0.003815 | 0.001034 |
| Parabacteroides |  |  | ✓ |  | ✓ |  | 0.005282 | 0.004781 |
| Peptostreptococcus |  |  |  | ✓ |  |  | 0.000095 | 0.000116 |
| Phascolarctobacterium | ✓ |  | ✓ |  | ✓ |  | 0.010859 | 0.000540 |
| Pseudocitrobacter |  |  | ✓ |  |  |  | 0.000137 | 0.000006 |
| Romboutsia |  | ✓ |  | ✓ |  |  | 0.017561 | 0.042739 |
| Roseburia | ✓ |  | ✓ |  |  |  | 0.019994 | 0.008515 |
| Ruminococcus | ✓ |  |  |  |  |  | 0.012914 | 0.007830 |
| Ruminococcus_gnavus_group |  |  |  | ✓ |  |  | 0.002605 | 0.003141 |
| Ruminococcus_torques_group |  | ✓ |  | ✓ |  |  | 0.010433 | 0.024406 |
| Salmonella |  |  | ✓ |  | ✓ |  | 0.000433 | 0.000053 |
| Shuttleworthia | ✓ |  | ✓ |  |  |  | 0.000296 | 0.000075 |
| Streptococcus |  | ✓ |  | ✓ |  | ✓ | 0.008792 | 0.022147 |
| UCG_002 | ✓ |  | ✓ |  | ✓ |  | 0.009133 | 0.003240 |
| UCG_003 | ✓ |  | ✓ |  |  |  | 0.000863 | 0.000349 |
| UCG_005 | ✓ |  | ✓ |  | ✓ |  | 0.001803 | 0.000300 |
| UCG_009 |  |  | ✓ |  | ✓ |  | 0.000106 | 0.000001 |
| Veillonella | ✓ |  | ✓ |  | ✓ |  | 0.003607 | 0.000972 |
| **Species** |  |  |  |  |  |  |  |  |
| Alistipes_obesi |  |  | ✓ |  |  |  | 0.000206 | 0.000076 |
| Bacteroides_caccae | ✓ |  | ✓ |  | ✓ |  | 0.001780 | 0.001290 |
| Bacteroides_finegoldii | ✓ |  | ✓ |  |  |  | 0.001426 | 0.000503 |
| Bacteroides_ovatus | ✓ |  | ✓ |  |  |  | 0.001049 | 0.000177 |
| Bacteroides_plebeius | ✓ |  | ✓ |  |  |  | 0.021144 | 0.002939 |
| Bacteroides_uniformis | ✓ |  | ✓ |  | ✓ |  | 0.006484 | 0.002567 |
| Bacteroides_vulgatus | ✓ |  | ✓ |  | ✓ |  | 0.025337 | 0.013157 |
| Bifidobacterium_longum |  | ✓ |  | ✓ |  |  | 0.006839 | 0.015081 |
| Bilophila_wadsworthia | ✓ |  | ✓ |  | ✓ |  | 0.000615 | 0.000058 |
| Blautia_faecis |  | ✓ |  | ✓ |  |  | 0.002975 | 0.007920 |
| Blautia_massiliensis |  | ✓ |  | ✓ |  | ✓ | 0.010201 | 0.030688 |
| Coprococcus_catus | ✓ |  | ✓ |  | ✓ |  | 0.001711 | 0.000826 |
| Dorea_longicatena |  | ✓ |  | ✓ |  | ✓ | 0.009030 | 0.027819 |
| Erysipelotrichaceae_UCG_003_bacterium |  | ✓ |  | ✓ |  |  | 0.006945 | 0.011885 |
| Faecalibacterium_prausnitzii |  |  | ✓ |  |  |  | 0.106483 | 0.060800 |
| Holdemania_filiformis |  |  | ✓ |  |  |  | 0.000098 | 0.000016 |
| Lachnoclostridium_edouardi | ✓ |  | ✓ |  | ✓ |  | 0.007192 | 0.001680 |
| Lachnospira_pectinoschiza | ✓ |  | ✓ |  | ✓ |  | 0.009789 | 0.001712 |
| Megamonas_funiformis | ✓ |  | ✓ |  |  |  | 0.044576 | 0.005034 |
| Parabacteroides_merdae |  |  |  | ✓ |  |  | 0.004234 | 0.004378 |
| Peptostreptococcus_stomatis |  |  |  | ✓ |  |  | 0.000096 | 0.000117 |
| Phascolarctobacterium_faecium | ✓ |  | ✓ |  | ✓ |  | 0.010997 | 0.000591 |
| Ruminococcus_bicirculans | ✓ |  | ✓ |  | ✓ |  | 0.002041 | 0.000071 |
| Streptococcus_mutans |  |  |  | ✓ |  |  | 0.000252 | 0.000700 |
| Veillonella_atypica | ✓ |  | ✓ |  | ✓ |  | 0.000731 | 0.000192 |
| Veillonella_dispar | ✓ |  | ✓ |  | ✓ |  | 0.001016 | 0.000211 |
| Veillonella_tobetsuensis | ✓ |  | ✓ |  |  |  | 0.000316 | 0.000078 |
